# Bilateral Ovarian Fibrothecoma Associated with Ascites, Bilateral Pleural Effusion, and Marked Elevated Serum CA-125

**DOI:** 10.1155/2013/189072

**Published:** 2013-02-03

**Authors:** Védi André Serges Loué, Eléonore Gbary, Sylvanus Koui, Bédi Akpa, Adélaide Kouassi

**Affiliations:** ^1^Department of Gynecology and Obstetrics, University Hospital of Cocody, 01 BP V13 Abidjan, Cote D'Ivoire; ^2^Faculty of Medicine, Félix Houphouët Boigny University of Cocody, 01 BP V 13 Abidjan, Cote D'Ivoire; ^3^Department of Visceral and Digestive Surgery, University Hospital of Cocody, Cote D'Ivoire; ^4^Department of Pathology, University Hospital of Treichville, Cote D'Ivoire

## Abstract

*Background*. The risk of ovarian cancer is increased in the association of ovarian tumor, ascites, and hydrothorax with the significant elevated tumor marker CA-125. However, this association can be observed in a rare clinical and benign pathological entity, that is Demons-Meigs' syndrome. *Objective*. To describe a rare case of Demons-Meigs' syndrome observed in our department. *Methods*. A black African woman of 35 years old, seventh gravida and fourth parous, underwent a total abdominal hysterectomy with bilateral salpingoophorectomy for large bilateral ovarian masses associated with significant ascites, bilateral pleural effusion, and particular highly elevated tumor marker CA-125 (1835 UI/mL) in a pronounced general alteration condition. *Results*. The postoperative course was uneventful characterized by a complete remission of hydrothorax and ascites with normal level of CA-125 three months after tumor excision. Histology of both masses revealed a bilateral ovarian fibrothecoma, a benign tumor of the ovary, thus confirming the diagnosis of Demons-Meigs' syndrome. *Conclusion*. The Demons-Meigs syndrome, although it strongly mimics the clinical picture of malignant metastatic ovarian cancer, remains a disease with benign prognosis after surgical tumor resection. This is a rare condition that must be known and recognized by practitioners to avoid unnecessary practices.

## 1. Introduction

The Demons-Meigs syndrome (DMS) in its initial typical form is a pathology that combines ovarian fibroma or fibrothecoma, ascites, and hydrothorax [1]. Complete, rapid, and definitive healing of the hydrothorax and ascites after surgical removal of the ovarian tumor is also a notable particularity of this syndrome [1]. This anatomoclinical entity, although not exceptional, is very rare. Recently, its definition has been expanded to all pelvic tumors (other than the ovarian fibroma or fibrothecoma) whether benign or malignant, associated with peritoneal effusion and/or pleural effusion without any suspected malignancy cell that regresses completely after tumor excision: one speaks then of Demons-Meigs pseudosyndrome [1–5]. The pseudosyndrome differs from the typical syndrome by histological nature and/or tumor location. Whatever the type of this syndrome (typical or atypical), the treatment is surgery and full recovery must always be observed [1, 4]. Despite the many cases reported in the literature, the etiopathogenesis of this syndrome is still not clearly defined [3, 4, 6].

We report the first documented case of a rare Demons-Meigs syndrome in our country associating bilateral fibrothecoma, ascites, and bilateral pleural effusion with significant elevation of the tumor marker CA-125.

## 2. Case Presentation

The patient, Z.A.S, a 35-year-old black African woman, housewife, seventh gravida and fourth para, with 5 living children, was received at the gastrointestinal surgery emergency of the University Hospital of Cocody (Abidjan, Cote D'Ivoire) on 25/05/2012 for abdominopelvic pain and abdominal mass. The beginning dates back to about a year by the occurrence of progressive abdominal distension in a context of amenorrhea and asthenia. Abdominopelvic pain is of recent onset (approximately 10 days prior to the patient admission) associated with a dry cough and difficulty breathing.

On admission, his general condition was altered. She was emaciated, dyspneic, and very asthenic. Conjunctivae were slightly colored, without jaundice. She had no edema or collateral venous circulation. Taking constant gave blood pressure = 10/06 Cmmhg, pulse = 120 bpm, breathe frequency = 22 cycles/min.

Physical examination showed an abundant ascites and a large abdominopelvic mass, diffuse, solid, mobile, and slightly painful in its entirety with increased pain in the pelvis. This mass extended from the pelvis to the *upper abdomen*. On vaginal examination, it was difficult to relate the mass to the uterus or annexes. Clinical examination also noted bilateral pleural effusion. Heart sounds were regular without breath.

**Figure 1:**
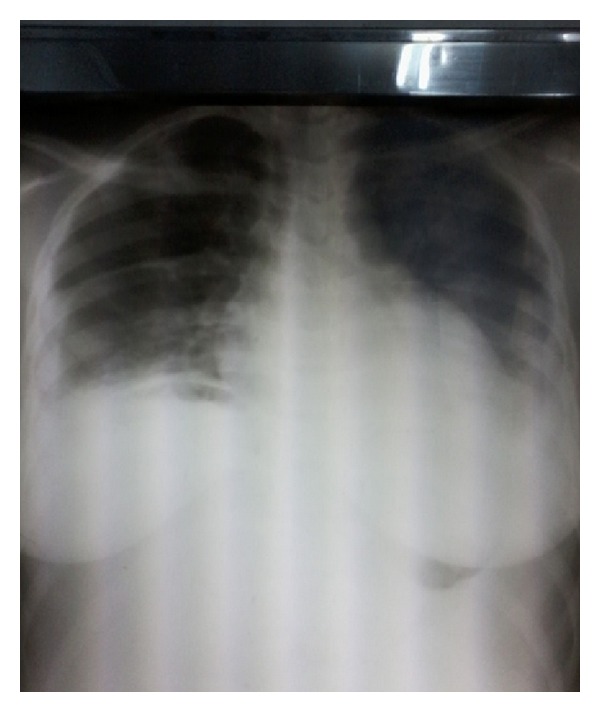
Chest radiograph showing a bilateral hydrothorax.

**Figure 2:**
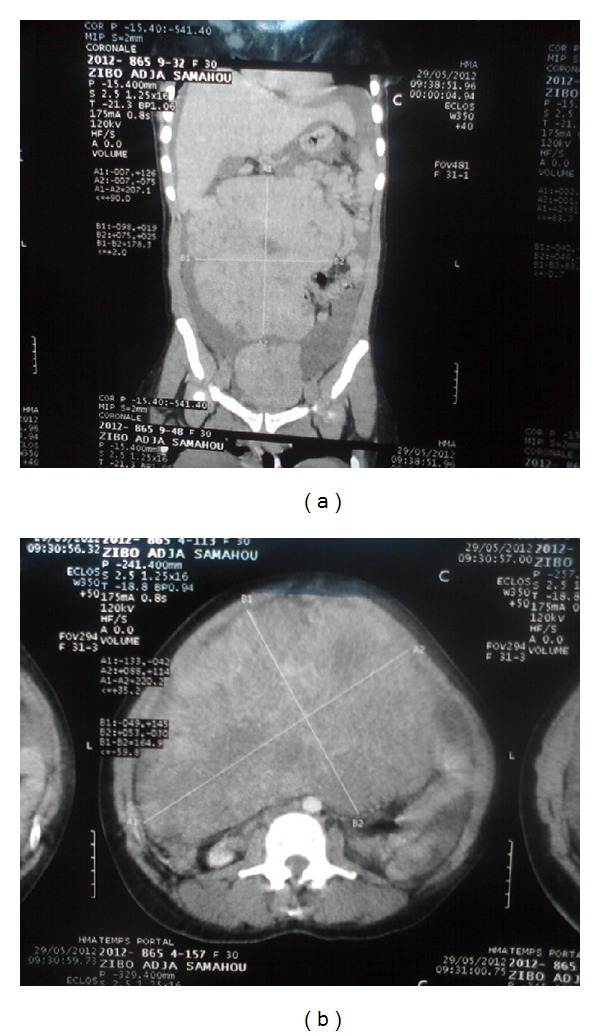
Abdominal CT scan showing two abdominopelvic tissue masses multilobulated.

The chest radiograph (Figure 1) performed in this patient showed a bilateral hydrothorax and the abdominal CT-scan (Figures 2(a) and 2(b)) visualized two tissue masses which one is abdominopelvic, multilobulated with multiple necrotic portions, large measuring 233 mm × 200 mm and the other located in the pelvis measuring 115 mm × 90 mm compressing the bladder adjacent dome without signs of invasion. Both masses are independent of the uterus, kidneys, and above mesocolic organs and coexist with abundant ascites without peritoneal nodulation suggestive of a bilateral ovarian tumor or a mesenchymal tumor.

Given the importance of dyspnea, pleural paracentesis (500 mL) and ascites (4.5 liters) were performed in emergency. Microscopic and chemistry study of both effusions was in favor of a transudate without any suspected malignancy cells.

The biological study of the liver and kidney was normal. A complete blood count was noted anemia to 7.9 g/dL. Tumor markers were measured: CEA (carcinoembryonic antigen) = 4.77 ng/mL (normal < 5 ng/mL); serum CA-125 level = 1835 IU/mL, more than 60 times above the normal superior limit (normal < 30 IU/mL); AFP (alpha-fetoprotein) = 8.1 ng/mL (normal < 20 ng/mL); ECG (electrocardiogram) was normal.

Given the importance of the alteration of the general condition and particular high elevated serum CA-125 with normal levels of other markers, the probable preoperative diagnosis of ovarian cancer was retained despite the lack of evidence of ovarian carcinoma at CT scan. After multidisciplinary consultation, an exploratory laparotomy was decided and performed on 03/06/2012 one week after the patient's admission.

**Figure 3:**
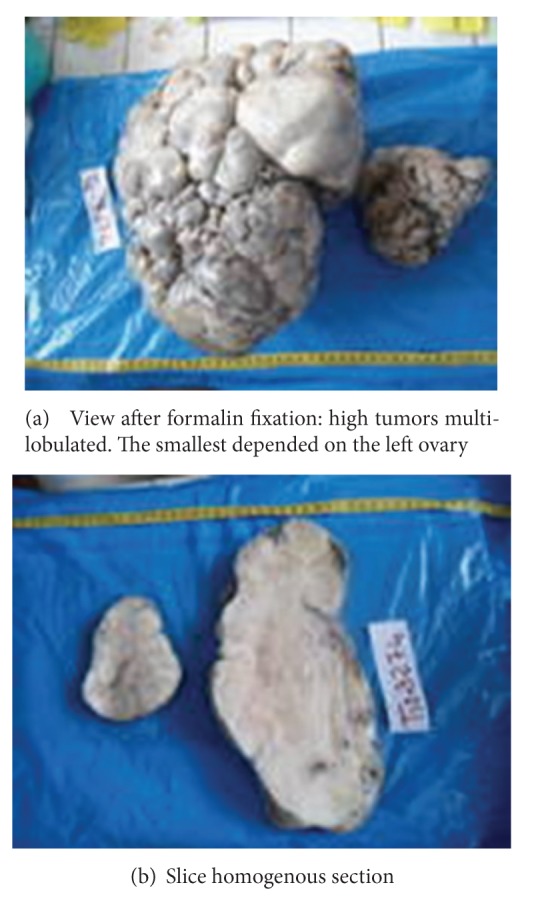
Macroscopic view of the masses.

Intraoperatively after a midline incision under and above the umbilicus, we find a 2.5 liter of ascites and two large ovarian masses (Figures 3(a) and 3(b)): a right ovarian tumor measuring 40 cm × 30 cm, multilobulated, solid, mobile, and a left ovarian tumor measuring 20 cm × 15 cm with the same characteristics like the first tumor and the left adnexal in subtorsion. Both ovaries were rolled by tumors. There were no lymphadenopathy or deep images of suspicious peritoneal carcinomatosis. Uterus, liver, gallbladder, and the spleen were macroscopically normal.

**Figure 4:**
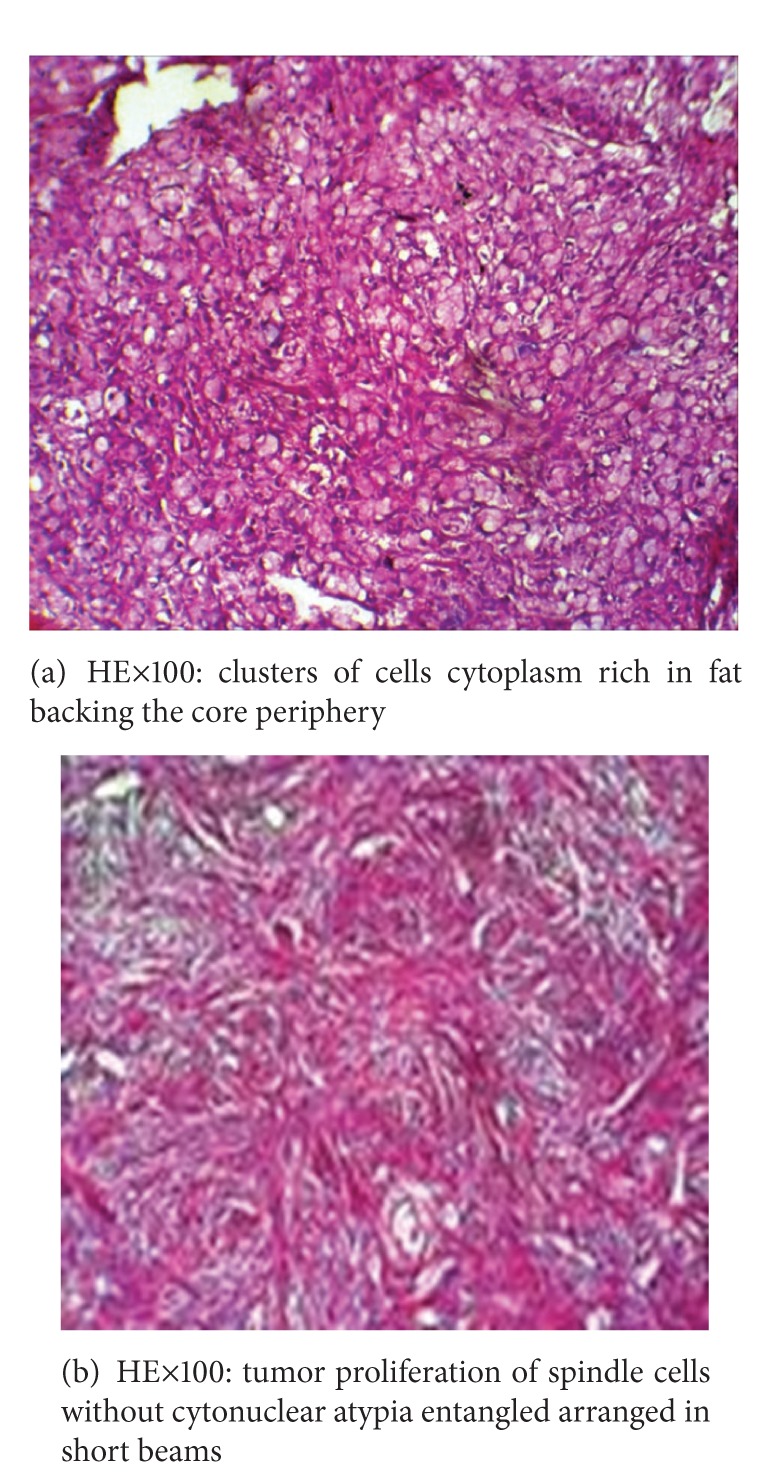
Histology of both surgical specimens in favor of ovarian fibrothecoma.

We performed a total hysterectomy with bilateral salpingoophorectomy. A blood transfusion of 1 liter of packed cells was required associated with analgesics. The postoperative course was uneventful without ascites reconstitution and the patient was discharged to J 12. Histological analysis of the two surgical specimens whose result is achieved 28 days later after the intervention concluded a double ovarian fibrothecoma which is a begnin tumor (Figures 4(a) and 4(b)).

The patient was revisited in consultation each month until the 3rd postoperative month. Clinical examination noted a lack of pleural effusion confirmed by chest radiography and absence of ascites by ultrasound postoperative control. The level of serum CA-125 was normal at last control. It then concluded at a Demons-Meigs syndrome.

## 3. Discussion

### 3.1. Frequency

The Demons-Meigs syndrome typically though not exceptional is a rare clinical and pathological [1]. Although its incidence is difficult to determine, it was noted that the ovarian fibroma represents 2–5% of all ovarian tumors and the incidence of DMS among patients with ovarian fibroma is estimated between 1% and 3% [3, 4]. In its unusual shape (pseudosyndrome), this syndrome is much rarer. This effect is probably largely underestimated when we know that in black Africa, most of the clinical findings are not yet published due mainly to the lack of certainty diagnostic due to a lack of reagents for histological study and/or because of its high cost.

Ovarian fibroma is a benign tumor most frequently observed in this syndrome in the order of 80–85% and may be pure and nonsecreting or associated thecoma elements (fibrothecoma) sometimes responsible for estrogen secretion. Thecomas and fibrothecomas represent 10% of cases [3, 4]. Fibrothecoma is benign ovarian stroma tumor, rare, and usually unilateral. We report here the first documented case of our country of a Demons-Meigs syndrome whose tumor is a giant bilateral ovarian fibrothecoma, complicated by the left adnexal subtorsion.

### 3.2. Etiopathogenesis

The prevalence of DMS begins to increase in the third decade and increases progressively to peak in the seventh decade. Although the syndrome may affect women of any age in both full genital activity (as our observation) and in prepubertal period [7]. The Demons-Meigs syndrome is more common in women with high socioeconomic status in the United States [3]. The extreme rarity of cases observed in sub-Saharan Africa cannot isolate the socioeconomic level as a risk factor of this syndrome.

Despite the many cases of the Demons-Meigs syndrome or pseudo-Meigs syndrome published in the literature, the mechanisms of formation of peritoneal and pleural effusions composing this syndrome remain unclear and speculative. Several theories attempt to explain the etiology of these two elements.

In DMS, ascites is present in 10–15% of cases and hydrothorax is found in only 1% of cases [3, 7–9]. Ascites is secondary either to a transudation to the surface of the tumor that is larger than the capacity of reabsorption of the peritoneum, or obstruction of the lymphatic vessels of the peritoneum by the ovarian mass, or to be a loss of protein and inflammatory response by increasing the permeability of neovessels [3–5]. An adnexal torsion and moisturizing action of sex steroids secreted by the ovarian tumor are also used as hypotheses, then the release of some mediators from the tumor leading to increased capillary permeability and be responsible for ascites [8, 9].

The hydrothorax is mostly unilateral and occurs most often on the right-sided (75%), rarely left-sided, but can be bilateral as in the observation that we present.

Indeed ascites once formed would spread through the diaphragm or via the lymphatic vessels of the foramen of Bochdalek in the chest cavity, more developed in the right than the left explaining the frequency of right-sided pleural effusion. Blockage of these lymphatics prevented the accumulation of pleural fluid and caused an increase in ascetic fluid. However, the minimum volume of ascites that can induce hydrothorax is not known [3, 5] thinking by other authors that there is no link between ascites and hydrothorax which would be two completely different symptoms [8, 9].

Ascitic and pleural fluids in Demons-Meigs' syndrome are similar in nature. It may be either transudative or exudative with a high frequency of exudate. The elevation of the tumor marker CA-125 is a confounding factor for that the CA-125 is increased in 80% of epithelial ovarian cancers but also in other malignancies and benign tumors [8, 10]. Let us remember that the CA-125 is not always increased in the Demons-Meigs syndrome and its increase was due to the irritation or inflammation caused by peritoneal ascites. In the observation that we present, bilaterality of ovarian fibrothecoma with bilateral pleurisy also associated with a particular high elevated serum CA-125 (more of 60 times the superior normal value) are particularities rarely found in the literature. However, there is no clear correlation between the volume of ascites, the serum CA-125 level, or the size and number of ovarian masses [5].

### 3.3. Diagnosis

The circumstances of discovery are variable and were the consequences of elements of the triad syndrome (ascites, abdominal mass, and hydrothorax). It can be a tension with abdominal bloating and weight gain or weight loss, respiratory distress associated with a cough or abdominal pain secondary to adnexal torsion as in the case we describe. A more or less pronounced alteration of the general condition can be observed. It may be a fortutious discovery during a routine gynecological examination, especially when the patient is asymptomatic.

Physical examination will typically find ascites, pleural effusion, and abdominal mass of variable importance. In the literature, most patients present an ovarian asymptomatic mass, solid, and unilateral, most often left-sided whose dimensions can be large [11, 12]. In our case, the woman was symptomatic with bilateral ovarian mass, solid with very large diameters.

Chest radiography confirmed hydrothorax while abdominopelvic ultrasound confirms the presence of a mass and ascites. The abdominopelvic CT scan specify the ovarian origin of mass and no signs of distant metastases. In our case, the CT scan did not diagnosis with certainty the organs which depend on the masses. Laparoscopic exploratory, which is still a luxury in our context, is reserved for masses of small dimensions. CA-125 can be increased but is not a diagnostic element because it is not specific to ovarian origin in the context of ascites; cytology of ascites and hydrothorax does not show any cells suggestive of malignancy [4].

In our case, such elevated rate of CA-125 (more of 60 times beyond the upper normal limit) with pronounced impairment of general condition and a tumor filling the whole abdominal cavity associated with serous effusions imitated advanced malignant ovarian tumor. In fact, any ovarian tumor with ascites and serous effusions should be considered malignant until proven otherwise histologically. Thus, the preoperative diagnosis of Demons-Meigs' syndrome or pseudosyndrome is difficult because of the clinic [5] as in our case; however, preoperative recognition is possible and ideal because it allows to limit the additional tests and prevent the realization of systematic surgical procedures in the treatment of ovarian cancer but useless in this benign pathology [3].

### 3.4. Treatment

Medical treatment is purely symptomatic and includes thoracocentesis and draining of ascites. Blood transfusion was necessary in our patient because of her preoperative anemia. Curative treatment is surgical by laparotomy in almost all cases [1–14]. Indeed laparotomy is the first compulsory treatment of ovarian tumors; it allows both the diagnosis of the tumor body, specifies the macroscopic characters of the tumor, research retroperitoneal lymph nodes and allows multiple biopsies for extemporaneous histological examination. Surgical treatment may be a bilateral salpingoophorectomy with or without hysterectomy in postmenopausal women or unilateral oophorectomy or unilateral salpingo-oophorectomy in young patients with the motherhood desire because of the low metastasis potential [3, 5]. In the case that we present, the obstetric history of the patient (5 living children), the laminated character of ovaries intraoperatively, the alteration of the condition of the patient and the significant elevated serum CA-125 which made us fear ovarian cancer, the bilaterality of the tumor, and because we have no extemporaneous histological examination, we performed a total hysterectomy with bilateral salpingoophorectomy. Ascites is not reconstituted since tumor excision and there was a complete disappearance of hydrothorax and normalization of the tumor marker CA-125 level three months after surgery. Indeed, the definitive and complete remission is a mandatory criterion sign of benignity which is even included in the definition of Demons-Meigs syndrome or pseudosyndrome [5, 11, 13] even if one case of recurrence 30 years after tumor excision has been reported [14].

## 4. Conclusion

The Demons-Meigs syndrome, although it strongly mimics the clinical picture of advanced ovarian cancer, remains a disease with benign prognosis after being properly managed. It should be considered systematically to all women presenting with a pelvic mass whatever the size or number associated with ascites and unilateral or bilateral hydrothorax with or without elevated CA-125 and in whom there is no concept of heart failure, nephrotic syndrome, or liver failure. The importance of general signs should not exclude the surgery. The histology of the tumor should confirm the syndrome but complete and definitive remission after surgery is also an important element of diagnosis. This is a rare condition that must be known and recognized by practitioners to avoid unnecessary practices.

## References

[1] Brun JL (2007). Demons syndrome revisited: a review of the literature. *Gynecologic Oncology*.

[2] Schmitt R, Weichert W, Schneider W, Luft FC, Kettritz R (2005). Pseudo-pseudo Meigs’ syndrome. *The Lancet*.

[3] Cissé C-T, Ngom P-M, Sangare M, Ndong M, Moreau J-C (2004). Ovarian fibroma associated with Demons-Meigs syndrome and elevated CA 125. *Journal de Gynécologie Obstétrique et Biologie de la Reproduction*.

[4] Cuillier F, David K, Tanguy M (2002). Un syndrome de Démons-Meigs réellement atypique. *Gynécologie Obstétrique & Fertilité*.

[5] Pire F, Wayembergh M, Wauters M, Renard N (2011). Syndrome pseudo-Meigs et goitre ovarien. *Gunaikeia*.

[6] Boufettal H, Zaghba N, Morad S (2011). Demons-Meigs syndrome: information on a new case and review of the literature. *Revue de Pneumologie Clinique*.

[7] Jiang W, Lu X, Zhu ZL, Liu XS, Xu CJ (2010). Struma ovarii associated with pseudo-Meigs’ syndrome and elevated serum CA 125: a case report and review of the literature. *Journal of Ovarian Research*.

[8] Mui MP, Tam KF, Tam FKY, Ngan HYS (2009). Coexistence of struma ovarii with marked ascites and elevated CA-125 levels: case report and literature review. *Archives of Gynecology and Obstetrics*.

[9] Loizzi V, Cormio G, Resta L, Fattizzi N, Vicino M, Selvaggi L (2005). Pseudo-Meigs syndrome and elevated CA125 associated with struma ovarii. *Gynecologic Oncology*.

[10] Coupe A, Baurain JF, Berliere M, Connerotte T, Whenham N, Duck L (2010). CA-125 and ovarian cancer: benefit, control and anxiety for patients or clinicians?. *Belgian Journal of Medical Oncology*.

[11] Salomon LJ, Lefebre M, Cortez A, Antoine J-M, Uzan S (2003). Fait clinique: goitre ovarien, une tumeur rare et particulière. *Journal De Gynécologie Obstétrique et Biologie de la Reproduction*.

[12] Roth LM, Talerman A (2007). The enigma of struma ovarii. *Pathology*.

[13] Su F, Cummings KW, Krigman H, Ranganathan P Meigs' sydrome: a rare cause of recurrent pleural effusion in scleroderma.

[14] Bretelle F, Portier MP, Boubli L, Houvenaeghel G (2000). Syndrome de Démons-Meigs récidivé. A propos d’un cas. *Annales De Chirurgie*.

